# Methane and the Paris Agreement temperature goals

**DOI:** 10.1098/rsta.2020.0456

**Published:** 2021-12-06

**Authors:** Michelle Cain, Stuart Jenkins, Myles R. Allen, John Lynch, David J. Frame, Adrian H. Macey, Glen P. Peters

**Affiliations:** 1 Centre for Environmental and Agricultural Informatics, School of Water, Energy and Environment, Cranfield University, Cranfield MK43 0AL, UK; 2 Atmospheric, Oceanic and Planetary Physics, Department of Physics, University of Oxford, UK; 3 Environmental Change Institute, School of Geography and the Environment, University of Oxford, UK; 4 New Zealand Climate Change Research Institute, Te Herenga Waka, Victoria University of Wellington, Wellington 6012, New Zealand; 5 CICERO Center for International Climate Research, Oslo, Norway

**Keywords:** climatology, atmospheric science, methane, climate, emission metrics, mitigation

## Abstract

Meeting the Paris Agreement temperature goal necessitates limiting methane (CH_4_)-induced warming, in addition to achieving net-zero or (netnegative) carbon dioxide (CO_2_) emissions. In our model, for the median 1.5°C scenario between 2020 and 2050, CH_4_ mitigation lowers temperatures by 0.1°C; CO2 increases it by 0.2°C. CO_2_ emissions continue increasing global mean temperature until net-zero emissions are reached, with potential for lowering temperatures with net-negative emissions. By contrast, reducing CH_4_ emissions starts to reverse CH_4_-induced warming within a few decades. These differences are hidden when framing climate mitigation using annual ‘CO_2_-equivalent’ emissions, including targets based on aggregated annual emission rates. We show how the different warming responses to CO_2_ and CH_4_ emissions can be accurately aggregated to estimate warming by using ‘warming-equivalent emissions’, which provide a transparent and convenient method to inform policies and measures for mitigation, or demonstrate progress towards a temperature goal. The method presented (GWP*) uses well-established climate science concepts to relate GWP100 to temperature, as a simple proxy for a climate model. The use of warming-equivalent emissions for nationally determined contributions and long-term strategies would enhance the transparency of stocktakes of progress towards a long-term temperature goal, compared to the use of standard equivalence methods.

This article is part of a discussion meeting issue ‘Rising methane: is warming feeding warming? (part 2)’.

## 1 Introduction

Methane (CH_4_) is the second most important anthropogenic contributor to present-day radiative forcing (RF), after carbon dioxide (CO_2_) and ahead of nitrous oxide (N_2_O), as shown in the intergovernmental panel on climate change (IPCC)’s Fifth Assessment Report [1]. As the Paris Agreement has a headline goal of limiting global warming to well-below 2°C and pursuing efforts to keep warming to 1.5°C, this article explores the potential role of CH_4_ in contributing to this temperature goal specifically. As a CH_4_ emission has a half-life of the order of a decade, its impact on RF and therefore temperature varies strongly with time after emissions occur. This is in stark contrast to the relationship between a CO_2_ emission and its impact on temperature, which remains relatively constant for hundreds of years after the emission (e.g. [2]). There is a near linear relationship between total CO_2_ emissions and CO_2_-induced global warming. Therefore, to stabilize anthropogenic CO_2_-induced warming, anthropogenic CO_2_ emissions must reach and remain at (or very near) zero [3]. The proportionality of cumulative CO_2_ emissions to the CO_2_-induced warming is the basis for the carbon budget concept [4,5]. Because of this link between CO_2_ emissions (or reduction thereof) and temperature, it has been reasonably straightforward for policymakers to incorporate the scientific insights of carbon budgets and the net-zero carbon emissions concept (where ‘carbon’ refers to CO_2_ only).

There is no such link between cumulative emissions and temperatures for CH_4_ [6], hence the need to address the question of CH_4_’s influence on temperature more explicitly. Typically, remaining carbon budgets make an adjustment for aggregated warming from non-CO_2_ emissions, so that the carbon budget is measured in CO_2_ only. There are fewer climate mitigation studies that model CH_4_’s impact on the climate explicitly and/or show its impacts independently, compared to the more widespread approach to report CO_2_-equivalent (CO_2_e) emissions only. By modelling CH_4_’s climate impact, time-varying effects or trade-offs can be investigated. Manning & Reisinger [7] use a method of comparing CH_4_ and CO_2_ based on the equivalence of their RF, based on the forcing-equivalent index introduced by Wigley [8]. Daniel *et al*. [9] show that the same annual CO_2_e emissions pathways lead to different temperature outcomes depending on if they are allocated to CH_4_ or CO_2_, and note that the flexibility of trading within a single-basket approach using GWP100 (Global Warming Potential over 100 years) comes at the cost of a more ambiguous RF outcome (GWP100 is a measure of the time-integrated RF from a pulse emission of a gas, relative to the same quantity for CO_2_, over 100 years). Reisinger & Clark [10] use a simple climate model and find that livestock’s contribution to global warming is 23% of the total warming, in contrast with its share of conventional CO_2_e emissions which is only 10–12%. Harmsen *et al*. [11] evaluate the impact of short-lived pollutant mitigation using integrated assessment models (IAMs) and note that while most countries have nationally determined contributions (NDCs) that cover all greenhouse gases, few specify targets for non-CO_2_ emissions. Their analysis showed that additional short-lived pollutant mitigation on top of the NDCs (including CH_4_) can reduce the global mean temperature in the short-term (by 0.03 to 0.15°C in 2040); however, it had little impact on the peak temperature for the 2°C pathways considered. The study confirmed previous work that showed CO_2_ mitigation is essential, but non-CO_2_ mitigation can offer an additional meaningful contribution (e.g. [7,12,13]). However, Harmsen *et al*. [14] point out that without specific CH_4_ policies, CH_4_ emissions will likely increase and therefore do need targeted consideration. Harmsen *et al*. [11] note that IAMs identify least-cost mitigation options based on GWP100 and not on the impact on global temperature of a mitigation option, which raises the question of whether an optimization based on temperature outcome would yield different results. Reisinger *et al*. [15] demonstrate different mitigation outcomes when comparing abatement costs allocated using GWP and global temperature-change potential (GTP), and Johansson *et al*. [16] found that using GWP100 cost 3.8% more than using an accurate method to assess trade-offs between mitigation of different greenhouse gases, which indicates a benefit to methods with improved accuracy.

To stabilize CH_4_-induced contribution to temperature (stable CO_2_-induced contribution to temperature being the result of achieving zero CO_2_ emissions), emissions of CH_4_ would need to decline at a rate of less than 1% per year on an ongoing basis [17]. This rate is derived from modelling the present state of the climate in a simple climate model. A different simple climate model shows that stable CH_4_ emissions henceforth would lead to atmospheric CH_4_ mole fractions reaching a steady state of just above 1900 ppb in 2100, compared to a present-day value of about 1800 ppb, an increase of around 6% [18]. Cutting those emissions with a linear decline by 30% in 2055, followed by stabilization, leads to a stable mole fraction of about 1200 ppb in 2100, a decrease of around a third [18]. One study found that the impact of CH_4_ changes on temperature can be expressed by equating each part per billion change in CH_4_ present in the atmosphere at 2100 compared to the present day to an emission of 0.27 ± 0.05 GtC [19].

In this article, we explore CH_4_’s role in a set of scenarios that achieve the Paris Agreement temperature goals using a simple climate model and emission metrics. Emissions metrics are used to place non-CO_2_ emissions on to a ‘comparable’ scale to CO_2_ emissions [1]. This is a standard method to compare different greenhouse gases when a climate model is not employed. There are well-documented shortcomings of the standard use of the GWP100, (e.g. [6–8,20,21]). If limiting the level of anthropogenic global warming is the goal, the importance of CH_4_ emissions increases as a temperature limit is approached, due to its relative forcing and short-lived behaviour [22]. The key obstacle we address here is that the common practice of aggregating greenhouse gases using GWP100-based ‘CO_2_-equivalent emissions’ does not adequately represent the role of CH_4_ on temperature, and that the problem is particularly acute for successful and aggressive mitigation pathways. Thus, aggregating all emissions on one CO_2_e scale with the standard application of GWP100 means that total aggregate CO_2_e emissions do not reliably relate to temperature trends or target. The IPCC’s most recent assessment report [23] does not recommend any specific metric, noting that the choice of metric depends on the purpose for which the emissions are being compared. The report highlights that expressing equivalence using GWP100 overstates the temperature impacts from a constant CH_4_ emission by a factor of 3–4 over a 20-year time horizon and conversely understates the impact of a new emission by a factor of 4–5 over the 20 years after it started. One study has shown that the range in the temperature response for scenarios consistent with the Paris Agreement temperature goal can be up to 0.17°C using GWP100 to determine the allocation of non-CO_2_ pollutants; more than a third of the remaining warming between current levels and 1.5°C [24]. Further, net-zero CO_2_e emissions defined using GWP100 (a common interpretation of Article 4 of the Paris Agreement) is not a necessity to achieve the temperature goal in Article 2 of the Paris Agreement [25,26]. If CH_4_ emissions are offset with CO_2_ removals to generate net-zero CO_2_e emissions using conventional GWP100, sustained declines in temperature arise [27]. A simple application of GWP100 in emissions-trading or carbon-pricing schemes between CO_2_ and CH_4_ would increase global warming on some timescales and decrease it on others [28].

A temperature goal can be met by limiting the cumulative total of long-lived emissions (i.e. a budget principle) and identifying a maximum future rate for short-lived pollutants [6]. This principle has been applied with the development of alternative emission metrics which account for the differences between long- and short-lived pollutants (e.g. [6]). GWP* [17,29–32] is an alternative application of GWP100 for CH_4_, with an equivalence between a step change in CH_4_ emissions and a pulse emission of CO_2_. The standard GWP100 concept would compare two pulse emissions. Refinements to GWP* [17,31,32] additionally account for the slower climate responses to historical changes in RF, and Smith *et al*. [32] demonstrate that GWP* relates RF from CH_4_ with that from CO_2_ directly. GWP* is effectively a simple climate model in a single equation and can be used to represent CO_2_-warming-equivalent (CO_2_-we) emissions (see Methods and Box 1). Other methods could be used in a similar way, e.g. CO_2_-forcing-equivalent emissions (CO_2_-fe), which defines equivalence based on CO_2_ emissions with the same modelled RF pathway [33]. Combined global temperature-change potential (CGTP) is another recent metric which allows short- and long-lived pollutants to be evaluated on a temperature basis [34]. It is not included in this analysis, as it is an approximation of monotonic changes to CH_4_ emissions, and therefore, because CH_4_’s effects are approximated as occurring instantaneously in parallel with that year’s emissions, it under-represents the inertia in the climate system and does not provide such a direct representation of temperature evolution over time from peak- and-decline scenarios. In such a peak- and-decline scenario, CGTP-based warming peaks too early and decays too sharply compared with a model simulation (e.g. fig 7.22 of [23]), though these divergences are considerably smaller than those associated with a GWP100-based approach.

The Paris Agreement contains numerous aims and goals, but the only quantified goal, with units, is the temperature goal articulated in Article 2, which states that the United Nations Framework Convention on Climate Change (UNFCCC) aims to strengthen the response to climate change, by ‘Holding the increase in the global average temperature to well-below 2°C above pre-industrial levels and pursuing efforts to limit the temperature increase to 1.5°C above pre-industrial levels’. This is normally interpreted [35] as a single aspirational goal requiring Parties to hold global temperatures to ‘well-below 2°C’ and as close to 1.5°C as possible. It is sometimes asserted that Article 4 (below) represents an additional, quantified goal, but the idea of balance is open to many possible interpretations [27]. Furthermore, the text of Article 4 makes it clear that Article 4 ought to be interpreted as being in service of Article 2:

In order to achieve the long-term temperature goal set out in Article 2, Parties aim to reach global peaking of greenhouse gas emissions as soon as possible, recognizing that peaking will take longer for developing country Parties, and to undertake rapid reductions thereafter in accordance with best available science, so as to achieve a balance between anthropogenic emissions by sources and removals by sinks of greenhouse gases in the second half of this century, on the basis of equity, and in the context of sustainable development and efforts to eradicate poverty.

The IPCC have not made a clear statement with reference to Article 4, but instead have stated that in pathways to limit warming to 1.5°C, CO_2_ emissions reach zero around 2050 and non-CO_2_ RF is reduced, but does not reduce to zero [26]. For example, a net-zero balance of all GHGs defined using GWP100 is not essential for a pathway to limit warming to 1.5°C, as over a quarter of the scenarios classified as achieving 1.5°C with a low overshoot in [36] to not reach this state by 2100 (see electronic supplementary material, figure S1). One study [37] concludes that using GWP100 to define a net-zero balance of all GHGs is unsuitable for policies aiming to meet a long-term temperature goal because it does not quantify temperature outcomes, and is particularly erroneous when applied to CH_4_ emissions reductions which are part of NDCs. Instead, the study suggests that the point of maximum GHG-caused RF should be classed as the point of reaching net-zero GHG, to provide a closer link to temperature outcomes. Defining net zero using CO_2_-we emissions would have very similar implications, with the sole difference being that a gradual decline in methane emissions and methane-induced radiative forcing is equated with net zero CO_2_-we emissions, this being what is required to prevent any further methane-induced increase in global temperatures. The choice of emission metric is a critical assumption when interpreting the ‘balance’ referred to in Article 4, with significant implications on the potential magnitude of CO_2_ removal required as a consequence. To examine CH_4_’s role in meeting the Article 2 temperature goal, and how that may relate to Article 4, we use a simple climate model to explore mitigation scenarios, followed by an analysis of the utility of different emissions metrics for the same purpose.

## 2 Methods

**Figure 1:**
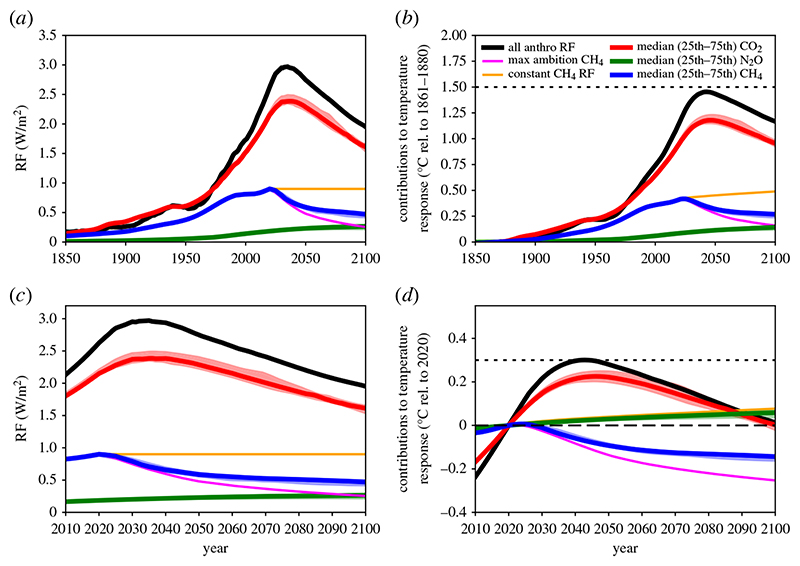
RF (*a,c*) and the resultant temperature response using the default FaIR2.0 model configuration (relative to 1860–1880 in (*b*) and relative to 2020 in (*d*)), for the median scenario (heavy lines) of the 1.5°C-compatible scenarios (see Methods). The range between the 25th and the 75th percentile scenarios is shaded. CO_2_, CH_4_ and N_2_O are shown separately, with the RF from tropospheric ozone included in the CH_4_-attributed RF and warming, as CH_4_ is the dominant driver of this signal. The total of all anthropogenic forcers (including those not shown individually) is shown in black. Additional scenarios are shown for CH_4_ only: a maximum ambition CH_4_ mitigation scenario (pink, described in Methods) and a scenario in which CH_4_ RF remains constant from 2020 onwards (orange). The dotted line shows 1.5°C above the 1860–1880 baseline temperature.

**Figure 2:**
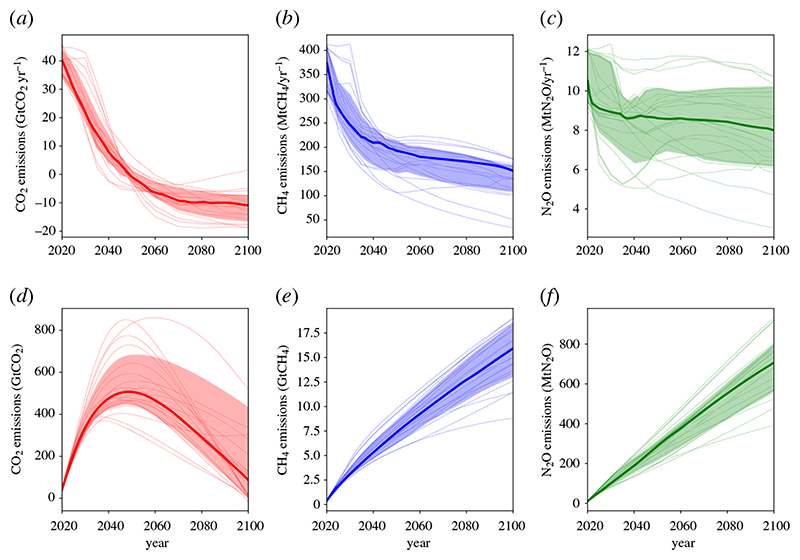
Emissions for scenarios compatible with a 1.5°C limit to warming (see Methods for details); (*a*,*b*,*c*) show annual emissions, and (*d*,*e*,*f*) show cumulative emissions since 2005. Heavy line shows the median and shading the interquartile range (see Methods).

Scenarios modelled in figure 1 are taken from the IPCC’s Special Report on the Global Warming of 1.5°C (SR15) [38] scenario database [36]. The CO_2_, CH_4_ and N_2_O emissions and RF are the 1.5°C-compatible model-scenario combinations which are consistent with present-day RF levels in each pollutant (using the method of [39]). The emissions of CO_2_, CH_4_ and N_2_O in these 22 scenarios are shown in figure 2. The RF attributed to CH_4_ here includes RF from tropospheric ozone, as this is primarily driven by CH_4_ emissions [40]. RF timeseries are extended to pre-industrial using the RCP8.5 RF timeseries prior to 2020 [41], where RCP8.5 RFs are scaled to match individual scenario RFs in 2020. The median and interquartile range of RF from the scenarios are calculated for each year to produce timeseries for figure 1. The ambitious CH_4_ scenario uses the most ambitious post-2020 CH_4_ mitigation scenario in the SR15 database (IMAGE3.0.1 IMA15-TOT model/scenario [42]) and is scaled to match the 2020 median CH_4_ RF from the range of 1.5°C-compatible CH_4_ mitigation scenarios. The constant CH_4_ RF scenario uses the same time history, with stable CH_4_ RF post-2020.

Temperature responses in figure 1 are calculated with the FaIRv2.0 simple climate model [43]. Total anthropogenic RFs are run through FaIRv2.0, along with separate runs where all RF is included except one component (e.g. total anthropogenic minus CO_2_ RF; total anthropogenic minus CH_4_ RF) following the work of [33]. Temperature contributions from individual components/scenarios are calculated by differencing full-anthropogenic and full-anthropogenic-minus-scenario temperature responses. Scenarios are shown relative to present day (2020) and relative to pre-industrial (1861–1880 baseline). Dotted lines in figure 1 show the Paris Agreement 1.5°C temperature limit for each reference period (0.3°C-above present day).

**Figure 3:**
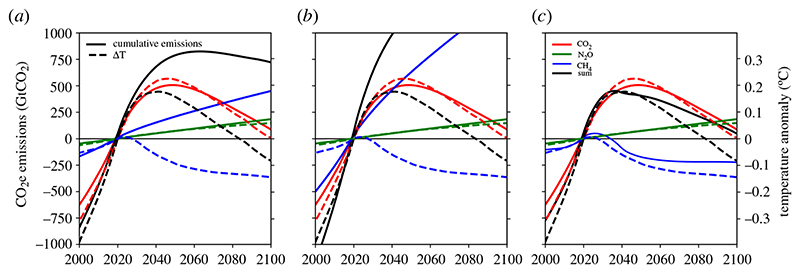
Solid lines show cumulative emissions of CO_2_ (red), CH_4_ (blue) and N_2_O (green), with each panel converted into CO_2_-equivalent using a different metric: GWP100 (*a*), GWP20 (*b*) and GWP* (*c*). Dashed lines show the temperature generated by the simple climate model (FaIR2.0) from the RF in the median scenario in figure 2 for each gas, and the sum of the three (black). A TCRE of 0.4°C/TtCO_2_ is used to scale the primary and secondary *y*-axes.

Figure 3 uses the median CO_2_, CH_4_ and N_2_O emissions scenarios corresponding to the median RFs in figure 1 and displays the cumulative CO_2_-equivalent emissions using GWP100, GWP20 and GWP* metrics, comparing them to temperature responses for each scenario, generated using FaIR2.0 as described for figure 1. GWP100 (28 for CH_4_ and 265 for N_2_O) and GWP20 (84 for CH_4_ and 264 for N_2_O) values were taken from [1]. GWP* emissions (E*) at time *t* are calculated using the updated formulation in [32], where the relative change in multidecade (20 years) CH_4_ emissions (*E*_CH4_) is used to estimate equivalent CO_2_ emissions: E*(*t*) = 128 x *E*_CH4_(*t*) – 120 x *E*_CH4_(*t*-20). Temperature responses (secondary axis) in figure 3 are scaled to the cumulative emissions (primary axis) using a Transient Climate Response to cumulative carbon emissions (TCRE) of 0.4°C/TtCO_2_, which is representative of the current climate system in FaIR2.0 [43].

Full descriptions of the metrics used in this paper can be found in [1,32].

## 3 Results

figure 1 shows the contributions to RF (*a*,*c*) and modelled temperature (*b*,*d*) from CO_2_, CH_4_ and N_2_O in the median and interquartile range of the 1.5°C compatible scenarios, relative to a pre-industrial baseline (*a*,*b*) and relative to 2020 (*c*,*d*). For CH_4_, a stable (constant) RF scenario is also shown (orange line). Given that CH_4_ emissions are currently increasing, and not declining as the mitigation scenarios do, the stable RF scenario is explored as a minimal CH_4_ mitigation scenario. If CH_4_ emissions were to continue rising, its RF and contribution to temperature would continue to rise. Nisbet *et al*. [44] note that if current CH_4_ trends continued, CH_4_ would likely reach 2400 ppb by 2100, and that atmospheric CH_4_ is currently between the RCP4.5 and RCP8.5 scenarios, which has 0.14 W m^−2^ higher RF at 2030 than the RCP2.6 scenario (which keeps warming below 2°C in 2100 with 66% probability).

Figure 1 shows the different roles of the three key greenhouse gases towards mitigating global warming, with key values shown in table 1. In the median scenario, CO_2_ adds 0.22°C to a global mean surface temperature between 2020 and the mid-century peak in temperature, taking CO_2_-induced warming to about 1.2°C. At around 2050, CO_2_ emissions become net-negative (figure 2) and CO_2_’s contribution to warming starts to decline. By 2100, CO_2_’s contribution to global warming has returned to present-day levels (marked by the dashed line in figure 1d). The interquartile range of scenarios (shaded) qualitatively shows a similar future.

In all the Paris-compatible scenarios shown, CH_4_’s contribution to warming peaks at the present decade (at around 0.4°C-above pre-industrial) as this is when CH_4_ emissions peak (blue and pink in figure 1). The subsequent reductions in CH_4_ emissions are sufficient to reduce CH_4_-attributed RF and temperature within a few decades. Considering the case where RF from CH_4_ is held constant into the future (orange), the temperature continues to increase beyond 2100. This indicates the climate’s longer timescale response to changes in RF (355 years in the model) and shows why some reductions in CH_4_ emissions (and therefore RF) would be needed even to stabilize CH_4_’s contribution to warming. Between 2020 and 2100, CH_4_ emissions decline 57–65% (interquartile range) in the individual scenarios shown, which reduces global warming by around 0.14°C over the same period (blue). If CH_4_ reductions were larger, this reduction in global warming would also be larger. It is unclear if the narrow range of CH_4_ reductions represents the maximum potential in these IAMs, or if deeper reductions are possible with the inclusion of additional demand- or supply-side reductions, or with higher costs. The maximum ambition scenario (pink) reduces CH_4_ emissions by 88% and is an outlier scenario which was specifically designed to maximize non-CO_2_ reductions [42]. The models are not independent of each other, and this consistency does not represent a ‘most likely’ future scenario [39]. It is simply the most cost-efficient mechanism for these models to avoid exceeding the 1.5°C temperature constraint, and is a constrained exploration of potential futures. Many models miss many mitigation options for CH_4_ (e.g. diet change) [38,42], and the experimental design of many studies (end-of-century targets) may give an unintentional preference for the use of CO_2_ removal over non-CO_2_ mitigation which has a shorter term effect on temperature [45]. Indeed, studies have found a strong trade-off between non-CO_2_ mitigation and CO_2_ mitigation in deep mitigation scenarios (fig. 1f in [46]; [47]).

It should also be noted that CH_4_ emissions to date are not showing signs of following these mitigation pathways. Atmospheric CH_4_ has been rising since 2006, likely driven by increasing emissions (potentially both natural and anthropogenic, from biogenic and fossil sources) [48]. Under the scenario with maximum CH_4_ reductions (pink), which includes more expansive CH_4_ mitigation options [42], temperature is reduced by around 0.25°C between 2020 and 2100. If CH_4_ RF remained constant over the rest of the twenty-first century (orange, which would be driven by gently declining emissions), it would add nearly 0.1°C on to its present-day contribution to warming. This is similar to the level of warming generated by the median scenario for N_2_O, in which N_2_O emissions reduce by about a third by 2100 (figure 2c). The temperature continues to rise after RF from CH_4_ stabilizes because the climate is still responding to past increases in RF and will do so for several hundred years [2,17].

These different scenarios for CH_4_ mitigation illustrate the importance of CH_4_ mitigation, given that the median scenario reduces temperatures by over 0.2°C compared to the stable CH_4_ RF scenario. This is similar to the temperature impact of the net CO_2_ removals over the same period. This can be explored with a simple comparison using TCRE as an estimate of how much warming is generated by cumulative CO_2_ emissions. Using a TCRE of 0.4°C/TtCO_2_ (as we use elsewhere in this study), this indicates that a reduction in temperature of 0.1°C (the difference between 2020 and about 2050 in figure 1d for CH_4_) equates to a net removal of about 250 GtCO_2_. By assuming CO_2_ removals increase linearly from zero in 2020 up to 2050, to remove 250 GtCO_2_ over this period would require 16 GtCO_2_ removals in 2050. If it took 50 years to remove the 250 GtCO_2_, this would mean reaching 10 GtCO_2_ removals in 2070. This is a substantial rate of CO_2_ removals. The potential for Direct Air Capture with Carbon Capture and Storage (DACCS) and Bioenergy with Carbon Capture and Storage (BECCS) could be a maximum of 5 GtCO_2_ per year according to one synthesis [49], noting several cited papers suggest higher levels. Given the feasibility concerns of large-scale CO_2_ removal, it is prudent to assess potential pathways with higher levels of CH_4_ mitigation [42].

Comparing the emissions of each greenhouse gas in the median scenario (and the interquartile range, shaded) in figure 2 with the resultant contribution to global warming in figure 1 illustrates the differences between long- and short-lived greenhouse gas emissions. The level of CO_2_ (red, figure 1d) and N_2_O (green, figure 1d) induced warming follows the same trend as the *cumulative* emissions trend (figure 2d,e). Long-lived gases drive temperatures upwards unless their emissions cease. The level of CH_4_-induced warming (blue, figure 1d) tracks the *annual* emission rate (figure 2b), with only a small component that is dependent on historical CH_4_ emissions. Therefore, CH_4_-induced warming can reduce if CH_4_ emissions reduce.

Table 1 summarizes the contributions to global warming from the median of the 1.5°C scenarios for CO_2_, CH_4_, N_2_O and the total of all anthropogenic forcings. The total includes forcings that are not included here individually, including aerosols which have a cooling impact, which is why the total contribution is in some cases lower than the sum of contributions from CO_2_, CH_4_ and N2O. At present, CH_4_ contributes just over a third of the total net anthropogenic global warming (relative to a pre-industrial baseline). This declines to less than a quarter at the time of peak warming in our median simulation and remains at that percentage to 2100, although the absolute contribution declines from 0.33°C to 0.26°C. Over the same period, N_2_O contribution rises in both absolute and relative terms, as it is long-lived and because emissions do not decline to zero, it accumulates in the atmosphere over these timescales. Table 1 shows clearly that CO_2_ is the dominant factor throughout, despite the very different temporal profiles from 2020 onwards (figure 1*b*).

## 4 Progress towards the Paris temperature goal

Emission metrics have been defined for different purposes. The GWP was originally formulated to compare the impact of two emission pulses using integrated forcing after 20, 100 or 500 years, noting that it was used as ‘a simple approach … to illustrate the difficulties inherent in the concept’ [50]. The GWP100 has been critiqued for not mapping to particular responses of interest (e.g. [22]), and alternative metrics have been designed, the GTP [51] being a common example. The GWP* was designed to provide a closer mapping between temperature and the cumulative effects of CO_2_ emissions, an area where GWP and GTP perform weakly [28]. A range of other metrics has also been developed (e.g. reviewed in [52]). In the context of the Paris Agreement and tracking progress towards emission targets, emission metrics could be used in different ways. Progress of countries towards the 1.5°C global warming limit could be evaluated based on comparing absolute emissions in a given year to a baseline (e.g. 2030 emissions relative to 1990), or cumulative emissions over a given time period (e.g. since 1990 or since a pre-industrial baseline). Depending on the comparison, the preferred metric might change, but in all cases, it is necessary to distinguish emissions from cumulative climate pollutants (those like CO_2_ and N_2_O with atmospheric residence times longer than 100 years) and from short-lived climate pollutants to evaluate the temperature response.

Since global warming is dominated by CO_2_ emissions, and CO_2_-induced warming relates linearly to cumulative CO_2_ emissions, tracking progress in terms of cumulative emissions is a natural development. It is scientifically possible to evaluate warming relative to a pre-industrial baseline, although this would introduce many political questions [53]. To quantify contributions to warming from different emissions requires a climate model, or if that is not feasible, then a proxy for a climate model. Some emission metrics act as a proxy for a climate model and can therefore approximate the warming generated by climate models, e.g. GWP*, CO_2_-fe or CGTP (as discussed in the introduction) to generate what can be referred to as ‘CO_2_-warming-equivalent’ emissions, to distinguish from conventional CO_2_e emissions which have an ambiguous relation to warming. Here, we explore the use of GWP* as a simple CO_2_-we metric. We also include GWP100 and GWP20, which are two common emission metrics that are a poor proxy for the temperature response. The key difference between warming-equivalent and conventional metrics is that the latter use a single exchange rate to convert CH_4_ emissions to CO_2_e emissions. Warming-equivalent metrics do not do this—they instead formulate an equivalence between a step change in CH_4_ emissions (positive or negative) and a one-off emission of CO_2_ (positive or negative). When CH_4_ emissions rise, they equate to a positive CO_2_-we emission of CO_2_, which gives approximately the same temperature change as the CH_4_ emissions. When CH_4_ emissions fall at a rate greater than required to stabilize CH_4_-induced warming, they equate to negative CO_2_-we emissions (and likewise, the same induced temperature change as the CH_4_ emissions).

As CO_2_-we emissions are linked to temperature, we show in figure 3 how cumulative CO_2_-we emissions relate to modelled warming, as a proxy for a climate model, using the median scenario. Cumulative CO_2_e emissions since 2020 are shown as derived using GWP100 (figure 3a) and GWP20 (figure 3b), for CO_2_ (red), N_2_O (green), CH_4_ (blue) and the sum of all three (black). The dashed lines show the temperature-change relative to 2020, due to each of the gases, from the model run of the median scenario (as shown in figure 1, except that figure 1 shows all anthropogenic forcings in black, and figure 3 shows the sum of CO_2_, N_2_O and CH_4_ only). This shows that cumulative CO_2_ emissions (solid red) follow a similar path to the warming that the model generates from those CO_2_ emissions (red dashed), with a good agreement using a TCRE of 0.4°C/TtCO_2_ to scale the primary and secondary y-axes (TCRE is the amount of warming per trillion tonnes of CO_2_ emitted). There is considerable uncertainty in the TCRE, but since the forcing–temperature relationship is linear, this simply scales the figure axis and does not affect our overall conclusions. For both GWP100 and GWP20, cumulative N_2_O emissions approximately follow the warming generated as well. N_2_O has a lifetime of over 100 years, so this similarity is expected. For CH_4_ (blue), the cumulative CO_2_e emissions do not relate to the warming those emissions generate (blue dashed), where an ever-increasing cumulative total corresponds to a reduction in warming contribution. This shows that framing targets solely in terms of GWP100 or GWP20 would be an inadequate measure of their contribution towards global warming or global cooling, and supports previous work e.g. [21,52].

Figure 3*c* shows cumulative CO_2_-we emissions defined using GWP* (identical to GWP100 CO_2_e emissions for N_2_O, as it is long-lived), which align better with the modelled temperature (dashed). For CH_4_, the GWP* (see Box 1) generates an equivalence based on approximating the RF that this CH_4_ timeseries would generate with CO_2_ emissions [32], and therefore the cumulative CO_2_-we emissions (blue solid) have a good agreement with the temperature anomaly (dashed blue).

## 5 Discussion

Methane is the only major greenhouse gas that has declining induced warming from 2020 to 2050 in these 1.5°C compatible mitigation scenarios (figure 1). Following continued CH_4_ reductions under the median scenario, CH_4_-induced warming in 2100 is approximately equal to its 1980 level. This does not mean that CH_4_ emissions are the same in 2100 as in 1980, as there is a lag between emissions and warming. CO_2_ and N_2_O emissions, even under ambitious mitigation scenarios, continue to cause further temperature increases beyond the present-day, as they are cumulative pollutants. For CO_2_, removals across the second half of the century mean that in these scenarios, CO_2_-induced warming declines from its mid-century peak and returns to 2020 warming levels by the end of the century. N_2_O’s contribution to temperature increases continues increasing up to 2100 (and beyond, not shown) despite modest emission reductions.

Meeting any goals for limiting global temperature, including Paris goals, inevitably means net-zero or net-negative CO_2_ emissions are required. The scale of net-negative emissions is dependent on the net temperature changes caused by non-CO_2_ mitigation and the climate response to net-zero CO_2_ emissions [3], as well as whether there is an overshoot of the temperature goal, which would need to be reversed. Net-zero CO_2_ emissions stabilize CO_2_-induced warming; net-negative CO_2_ emissions returns the temperature to some past level of warming, with the exact level depending on the behaviour of the climate system [3]. Therefore, the requirement for net-negative CO_2_ emissions largely depends on how much CO_2_ has been emitted at the time of reaching net-zero CO_2_ emissions (e.g. [45]). In this sense, the so-called ‘race to net-zero’ for CO_2_ emissions is a race to stabilize temperature. CH_4_ being short-lived means that a race to net-zero CH_4_ emissions would undo past warming; the CH_4_-induced warming would reduce long before net-zero CH_4_ emissions were reached. To produce the temperature stabilization that we would get from net-zero CO_2_ emissions would require reducing CH_4_ emissions by around 0.5% per year.

The necessity of taking different approaches to CO_2_ and CH_4_ (and, more broadly, longer and shorter lived GHGs) to anticipate temperature changes raises interesting questions around how we assess different contributions to climate change and set emission targets to contribute to the overarching temperature-based goals.

From a ‘warming-equivalent’ perspective, a net-zero CO_2_ target might be suggested as directly equivalent to requiring CH_4_ emissions to reduce by only 0.5% each year, from whatever their current rate. This gives a consistent treatment to CO_2_ in terms of overall temperature-change expectations, but has also been argued as grand-fathering the CH_4_ emission rights [54], as in this case past emissions lead to the entitlement of continued emissions [55]. Questions over how to combine and compare different gases in order to achieve overarching climate goals thus come down to whether to base our concepts of ‘equivalence’ on overall temperature outcomes or on the contemporary act of emitting a greenhouse gas.

Most approaches thus far frame climate policy as a series of decisions to emit pulses of greenhouse gases, compatible with the design of the Kyoto Protocol and emission trading systems such as the EU-ETS. The focus is thus on the act of emitting, and the ‘equivalence’ between gases is based on some pre-defined measure (i.e. the GWP100) of the impact each individual emission would have compared to not emitting it. But the notion that ‘every tonne emitted’ is a discrete action belies the way in which many policy decisions are actually made, and frameworks based only on contemporary or future emissions are fundamentally insufficient to address any issues around overall global warming or goals relating to this.

If future GHG emissions are presented using CO_2_-we, the impact on relative temperature change will be more accurately reflected for each GHG and across the whole time period (figure 3c). The reduced temperature from abating CH_4_ versus not abating CH_4_ thus becomes transparent and can be compared directly with the impacts on temperature from abating other GHGs. figure 1 shows that the difference between holding RF from CH_4_ constant from 2020 (orange line) and applying maximum ambition on CH_4_ (pink) leads to a difference of nearly 0.2°C in 2050. This means that the climate benefits foregone by not abating CH_4_ could lead to nearly as much warming as will be expected from CO_2_ emissions over this period (red). figure 1 also shows that taking a start point of 2020 will show that CH_4_ reductions lead to a declining temperature contribution (figure 1d), but this is entirely depending on the choice of start date. A pre-industrial base year (figure 1b) still shows an overall warming from CH_4_, it has just declined from a peak around 2020.

The choice of base years applies equally to emissions of other gases, where 1990 or 2005 are often used as a recent and practical base year in climate policy, yet means that any contribution to global temperature increase made by emissions occurring before this year is dropped from explicit policy consideration. The Paris Agreement being primarily framed in terms of setting overall warming limits above pre-industrial levels implies some significance to this total amount, and not just what can be avoided by reducing current and future emissions.

To account for the full history of warming, from pre-industrial levels, requires different treatment for CO_2_ and CH_4_. For CO_2_, a total carbon budget of the accumulated CO_2_ emissions from pre-industrial times to the given year maps to the CO_2_-induced warming (e.g. total CO_2_ emitted by the time net-zero CO_2_ emissions is achieved). CH_4_-induced warming follows more closely annual CH_4_ emissions, not cumulative CH_4_ emissions. Further, the current carbon budget approach lumps all non-CO_2_ emissions together, consisting of short- and long-lived species with warming and cooling effects [51], which provides minimal motivation for mitigating different components of non-CO_2_ emissions. Potential methods to disaggregate the non-CO_2_ warming include: a CO_2_-we budget, as accumulated CO_2_-we emissions to date are analogous to accumulated CO_2_ emissions [36]; or a specified upper level of RF (or global warming) allocated to CH_4_ or other non-CO_2_ emissions (which echoes the framing presented in the IPCC’s Special Report on 1.5°C [26]). Both of these methods would be broadly applicable to the temperature goal in the same way the carbon budget is.

CO_2_-we emissions (for components like CH_4_) work the same way as cumulative CO_2_ emissions: they summarize warming from the beginning of the analysis period. By design, cumulative CO_2_-we emissions accurately reflect the warming over the period considered, just as cumulative CO_2_ emissions reflect the CO_2_-induced warming over the period considered. Neither reflects warming from before the timeseries starts. To include warming from earlier periods, the analysis of either cumulative CO_2_ or cumulative CO_2_-we emissions can be started earlier. Historic warming (of about 1.2°C) is the dominant factor leading to 1.5°C (e.g. [56]), given that we are only a few tenths of a degree from the 1.5°C limit (https://www.globalwarmingindex.org/). CO_2_-we emissions simply generalize this point to allow us, cogently, to include shorter lived greenhouse gases into the cumulative emissions concept, which is not possible with the conventional use of the GWP100 metric.

Assessments based only on contemporary annual or future projected emissions will mask the fact that cumulative CO_2_ emissions result in the vast majority of global warming globally (figure 1b) and for developed countries [57], and that a significant amount of warming caused by the biggest historical emitters is from emissions that occurred before even 1990: US (25% of total cumulative CO_2_ emissions), EU28 (22%) and China (13%) (OurWorldInData.org). In order to accurately evaluate contributions towards climate change, a consistent approach to temperature should be applied to both CO_2_ and CH_4_, with the same base year. Using CO_2_-we, temperature effects can be explored over any period of interest without the need to run a multi-component climate model. The concept of relating emissions targets to historical contribution to temperature is not new and has been proposed to the UNFCCC by Brazil [58]; a discussion of this context can be found in ref [57]. Different approaches to equitable mitigation have been explored using models, e.g. [59], and historical responsibility is one key element of consideration.

Even if we did not consider it important to incorporate the historical perspective, we might still want to reconsider whether like-for-like weighted emissions are an appropriate or optimal way of judging future impacts or setting future targets, given the limitations raised above and the potential for new versions of equivalence (i.e. ‘warming-equivalence’) to be applied even with contemporary baselines. Conventional ‘equivalent emission’ approaches will inevitably lose a clear link with overall temperature outcomes. ‘Warming-equivalence’ suggests a new way of conceptualizing emissions and, potentially, setting or evaluating emission targets, with a direct link to temperature outcomes. It could therefore also provide a basis for linking Article 4 (about emissions) with Article 2 (about temperature).

However, in doing so, it treats long- and short-lived gases separately and can result in different valuations for any given CH_4_ emission depending on its context within a wider emission series, because that context is essential to understand the impact on temperature. This has led to questions of how to apply warming-equivalence methods fairly [54]. Given the differences between long- and short-lived gases, it is not possible to treat individual emissions as directly equivalent and achieve the same temperature outcomes because the warming from short-lived species is temporary while that associated with long-lived species is permanent. Conversely, if we assess responsibility or set requirements based primarily on temperature outcomes, we must inevitably end up with different treatments and targets for different gases. Questions over historical responsibility and appropriate share of mitigation effort will still need to be resolved under either approach. We suggest the focus on the direct equivalence of contemporary emissions only (and universal targets based on this) obscures some of these points, and the challenges in linking conventional emission-equivalence to overall global warming remain underappreciated in many research and policy contexts and are worthy of further exploration.

## 6 Conclusion

The results presented here help to inform how we can assess whether NDCs would lead to achievement of the Paris temperature goal based on the best available science. If the assessment of progress towards a temperature limit of 1.5°C above pre-industrial temperature (without running a climate model) is the aim, then a metric which acts as a proxy for contribution to temperature will be needed to accurately represent CH_4_. The use of conventional GWP100 hides progress towards the temperature goal, as the amount of warming generated by CO_2_e emissions is ambiguous (e.g. [24,25,27]). The use of GWP20 reflects the integrated RF from a CH_4_ emission over the initial 20 years, compared to that of CO_2_, and is unsuitable for use over time horizons further than 20 years. If an equivalence was to be placed on CH_4_ and CO_2_ based on GWP20, this would place a high value on reducing a CH_4_ emission, despite the fact that in the long term, the nominally equivalent CO_2_ emission would have a much greater warming effect [28]. If a ‘net-zero’ scenario for all greenhouse gases were defined using GWP100 or GWP20, the temperature outcome would depend on the component gases (e.g. [9,60]). For example, if ongoing CH_4_ emissions were offset by CO_2_ removals, as is often assumed (e.g. [27,61]) then maintaining this net-zero scenario would cause temperatures to decline over time—after a couple of decades if using GWP20, and after a century if using GWP100 in the example shown by Allen *et al*. [28]. If, however, CH_4_ removals were used to offset CO_2_ emissions, e.g. as discussed in [62], then the temperature would increase while this net-zero scenario were maintained in the long term (again, after a couple of decades if using GWP20 and after a century using GWP100). This latter scenario, using GWP100 or GWP20, could be inconsistent with the temperature goal of the Paris agreement, if the trend caused temperatures to exceed ‘well-below 2°C’.

In summary, using conventional GWP100 for defining net-zero CO_2_e emissions for the Paris Agreement could result in a state of sustained, nominal net-zero emissions being associated with long-term warming or long-term cooling, which would be exacerbated by using GWP20. Use of a ‘warming-equivalent’ metric to define net-zero leads, by construction, to net-zero being associated with approximately stable temperatures. The ambition level of such a target would therefore be defined by the cumulative long-lived emissions at the time of net-zero and a measure of the short-lived contributions to RF at and over the decades prior to that time. This accounts for historical contributions and allows contributions from different emissions to be assessed and targeted in an explicit manner. Note that we are not advocating for any particular metric-defined net-zero target by explaining the implications of different metric-defined targets.

Here, we have shown that GWP* can be used to accurately represent the warming arising from 1.5°C scenarios.

As Article 4.2 of the UNFCCC states that ‘calculations of emissions by sources and removals by sinks of greenhouse gases (…) should take into account the best available scientific knowledge, including (…) of (…) the respective contributions of such gases to climate change. The Conference of the Parties shall consider and agree on methodologies for these calculations at its first session and review them regularly thereafter … ‘, we argue here that CO_2_-we emissions can be a useful tool for evaluating the effects of implementing NDCs on limiting global warming, as a simpler alternative to a climate model. It can therefore also be used to compare two potential mitigation pathways to show which one results in the lowest temperature outcome. Targets expressed as CO_2_-we emissions would allow short- and long-lived greenhouse gases to be brought into a single-basket approach, or to evaluate the sum total ambition of a two-basket approach. Because CO_2_-we emissions can be calculated directly from CO_2_e emissions reported using GWP100, their use in policy and target-setting is fully consistent with recent reporting decisions by the UNFCCC, but, crucially, require long-lived gases to be specified separately from total aggregate CO_2_e emissions in NDCs and long-term mid-century strategies. Agreement on separate reporting and target-setting for these cumulative pollutants would be a straightforward decision for the UNFCCC and would significantly enhance the transparency of stocktakes of progress to any long-term temperature goal.

## Supplementary Material

Electronic supplementary material is available online at https://doi.org/10.6084/m9.figshare.c.5680387.

S1

## Figures and Tables

**Table 1:** Contributions to global warming from CO_2_,CH_4_,N_2_O and all anthropogenic emissions (including those emissions not explicitly included in this table) for the median 1.5°C scenario, relative to a 1860–1880 baseline and a 2020 baseline, derived using FaIR2.0. Note that aerosol and F-gases are not shown separately, but do contribute to the ’all anthro’ category.

gas	contribution to global warming since 1860–1880 baseline			contribution of each gas to global warming relative to 2020	
	2020 (°C)	2046 (peak warming) (°C)	2100 (°C)	2046 (peak warming) (°C)	2100 (°C)
CO_2_	+0.95	+1.18	+0.96	+0.22	+0.00
CH_4_	+0.41	+0.33	+0.26	-0.08	-0.14
N_2_O	+0.08	+0.11	+0.14	+0.03	+0.06
all anthro	+1.15	+1.45	+1.17	+0.30	+0.01

## Box 1 GWP* and CO_2_-we

For CH_4_, the equation to calculate CO_2_-warming-equivalent (CO_2_-we) emissions using GWP*, denoted E*(*t*), is

$$E^{*}(t)=128\times E_{\text{CH}4}(t)-120\times E_{\text{CH}4}(t-20)$$

where ECH_4_ is CH_4_ emissions in tCH_4_ per year, and the coefficients include GWP100 values from [1].

Alternatively, to use CH_4_ emissions already in CO_2_e (E100) based on GWP100 from [1], the following numerically identical equation can be used:

$$E^{*}(t)=4.53\times E_{100}(t)-4.25\times E_{100}(t-20).$$

Emissions are used from years t (the year for which CO_2_-we emissions are being calculated) and from *t-20* (20 years prior). This allows the metric to represent the impact of new emissions (which cause strong additional warming), stable emissions (minimal additional warming) and reducing emissions (which reverses some past warming).

Equations in this box are from [32].
