# Supplementary material for: Methane and the Paris Agreement temperature goals
Source: Philos Trans A Math Phys Eng Sci. Author manuscript; Available in PMC 2022 Jan 24. (PMC8646145; doi:10.1098/rsta.2020.0456)

Supplementary information for: Methane and the Paris Agreement temperature goals

Michelle Cain[1*], Stuart Jenkins[2], Myles R. Allen[3], John Lynch[4], David J. Frame[5], Adrian H. Macey[6], Glen P. Peters[7]

[1]*Centre for Environmental and Agricultural Informatics, School of Water, Energy and Environment, Cranfield University, Cranfield, MK43 0AL ORCID: 0000-0003-2062-6556*
[2]*Atmospheric, Oceanic and Planetary Physics, Department of Physics, University of Oxford, UK. ORCID: 0000-0003-2284-0302*
[3]*Environmental Change Institute, School of Geography and the Environment, University of Oxford, UK and Atmospheric, Oceanic and Planetary Physics, Department of Physics, University of Oxford, UK. ORCID: 0000-0002-1721-7172*
[4]*Atmospheric, Oceanic and Planetary Physics, Department of Physics, University of Oxford, UK. ORCID: 0000-0001-7863-1767.*
[5]*New Zealand Climate Change Research Institute, Te Herenga Waka, Victoria University of Wellington, Wellington 6012, New Zealand ORCID: 0000-0002-0949-3994*
[6]*New Zealand Climate Change Research Institute, Te Herenga Waka, Victoria University of Wellington, Wellngton 6012, New Zealand. ORCID: 0000-0002-6963-893X*
[7]*CICERO Center for International Climate Research, Oslo, Norway ORCID: 0000-0001-7889-8568*

Fig S1: A box plot showing the 0, 25, 50, 75, and 100 percentiles for the net-zero GHG emissions year, as defined using GWP100, for different classifications of scenarios in the IPCC Special Report on Global Warming of 1.5C (SR15). The scenario classifications are as described in SR15. The scenarios end in 2100, and the box plots that are cut off in 2100 mean that the remainder of the distribution occurs after 2100. The 'marker' scenarios are the four illustrative pathways used in SR15, noting that one does not reach net zero GHG emissions by 2100 (The 'Low Energy Demand' scenario). Both the '1.5C low overshoot', and the combined group of '1.5C no and low overshoot' have over 25% of scenarios which do not reach net-zero GHG emissions, defined using GWP100, by 2100.

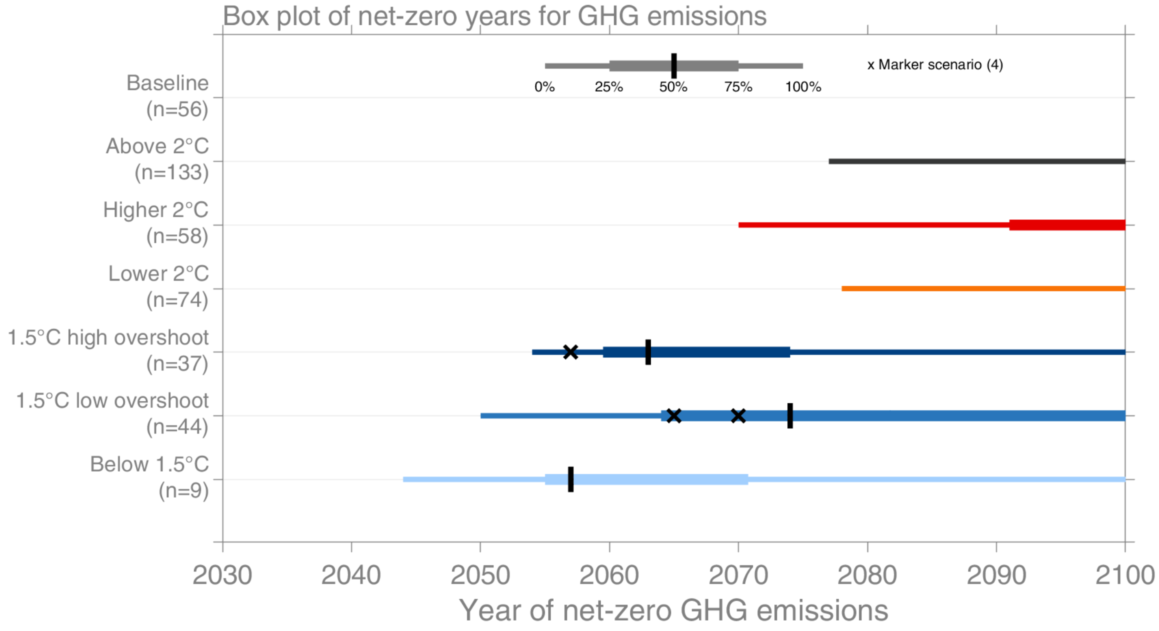

Supplement: S1 [file EMS140563-supplement-S1.pdf]
